# Atomic‐Scale Insights into Yttrium‐Induced Grain Boundary Structure Modification in Al_2_O_3_

**DOI:** 10.1002/advs.202515350

**Published:** 2025-12-22

**Authors:** Jingyuan Yan, Tatsuya Yokoi, Yuuki Nakano, Shun Kondo, Bin Feng, Naoya Shibata, Katsuyuki Matsunaga, Yuichi Ikuhara

**Affiliations:** ^1^ Institute of Engineering Innovation The University of Tokyo Tokyo 113‐0032 Japan; ^2^ Advanced Institute for Materials Research (WPI‐AIMR) Tohoku University Sendai 980‐8577 Japan; ^3^ Department of Materials Physics Nagoya University Nagoya 464‐8603 Japan; ^4^ PRESTO Japan Science and Technology Agency Kawaguchi 332‐0012 Japan; ^5^ Nanostructures Research Laboratory Japan Fine Ceramics Center Nagoya 456‐8587 Japan

**Keywords:** atomic structure modification, ceramics, grain boundary, isovalent segregation, NNP‐based MCMD calculation

## Abstract

Impurity segregation at grain boundaries (GBs) often induces structural transformations at the atomic level and significantly influences materials’ properties, underscoring the importance of understanding the underlying mechanisms of GB segregation at the atomic scale. Here, the atomic structure of a Y‐segregated ∑13(10$\bar{1}$4)/[$\bar{1}$2$\bar{1}$0] GB is thoroughly studied in α‐Al_2_O_3_ via scanning transmission electron microscopy and Monte Carlo and molecular dynamics simulations based on a neural network potential. It is found that the Y segregation at the GB involves not only the simple substitution of Y for Al atoms but also a structural adaptation with a change in GB atomic density. Such a change alters the local bonding environment at the GB so that the absolute excess volume is minimized for the lowest‐energy structure. This study offers a new insight into the atomic‐scale mechanism of GB structural transformation.

## Graphical abstract

Using advanced STEM characterization combined with NNP‐based MCMD calculations, it is revealed that Y segregation at ∑13 Al_2_O_3_ GB involves not only simple substitution of Y for Al atoms but also structural adaptation with a change in GB atomic density, leading to minimized excess volume and lowest GB energy. This work deepens the understanding of GB segregation at the atomic scale.

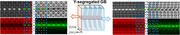

## 1 Introduction

Grain boundaries (GBs) have different atomic structures and bonding environments from the bulk, depending on their crystallography, and often have crucial impacts on macroscopic material properties, such as mechanical, thermal, electrical, and optical properties. Due to large excess volume and weak bonding, GBs often serve as natural sinks of foreign atoms with low solid solubilities in the bulk.^[^ ^1^, ^2^ ^]^ Such segregation can induce changes in atomic and electronic structure at GBs, leading to substantial influences on materials properties.^[^ ^3^, ^4^, ^5^ ^]^ A sophisticated control of GB segregation is thus essential to obtain desired microstructure and properties, as investigated in the framework of GB engineering.^[^ ^6^, ^7^, ^8^, ^9^, ^10^ ^]^ However, predicting the atomic structure of segregated GBs remains highly challenging due to the complexity of GB structure, atomistic properties of segregation, and interaction between solute and different dopants,^[^ ^11^, ^12^, ^13^ ^]^ etc. For example, as the simplest form of segregation, isovalent segregation usually simply substitutes the host atoms, without significantly changing the local electric field and coordination.^[^ ^12^, ^14^, ^15^, ^16^ ^]^ Nevertheless, since the dopants usually have different atomic sizes compared to the hosts, such simple substitution would result in changes in the local elastic field and strain level,^[^ ^17^, ^18^ ^]^ and complex defect structures could form.^[^ ^19^, ^20^ ^]^ These situations make it difficult to predict structure transformation even in the case of isovalent segregation, leaving mysteries about the atomic‐scale mechanisms of the segregation processes. On the other hand, there are some computational studies that suggested that the atomic density at GBs, which is defined by the number of atoms in the boundary core, could be a key factor in determining energetically favorable atomic structures of GBs,^[^ ^21^, ^22^ ^]^ which provide new insights into the structure transformation during GB segregation. But such a theory remains experimentally unverified, and the significance of such an effect has yet to be conclusively demonstrated.

In this research, we provide direct evidence for the isovalent segregation resulting in a change in GB atomic density. The ∑13(10$\bar{1}$4)/[$\bar{1}$2$\bar{1}$0] α‐Al_2_O_3_ GB was chosen as a model system since it is representative of the general GBs and promising for both experimental and computational studies.^23^ Besides, its suitable interfacial energy and local structural difference highlighted it as an excellent subject for studies on GB segregation. Exploring the underlying mechanism for different segregation behaviors would provide important knowledge for the GB segregation theory. Specifically, we directly characterized the GB's atomic structure of a Y‐segregated ∑13(10$\bar{1}$4)/[$\bar{1}$2$\bar{1}$0] α‐Al_2_O_3_ GB by scanning transmission electron microscopy (STEM). The observed image was well reproduced by Monte Carlo and molecular dynamics (MCMD) simulations based on our neural network potential (NNP). It was found that the GB‐structure transformation induced by the isovalent Y segregation is not a simple substitution. Instead, to compensate for the size effect of the larger substituting dopant, GB atom density was changed, and the new structure preserved the compactness of the O sublattice in pure GB and exhibited a different structural period, which helped to minimize GB excess volume and resulted in the lowest GB energy.

## 2 Results and Discussion

**Figure 1:**
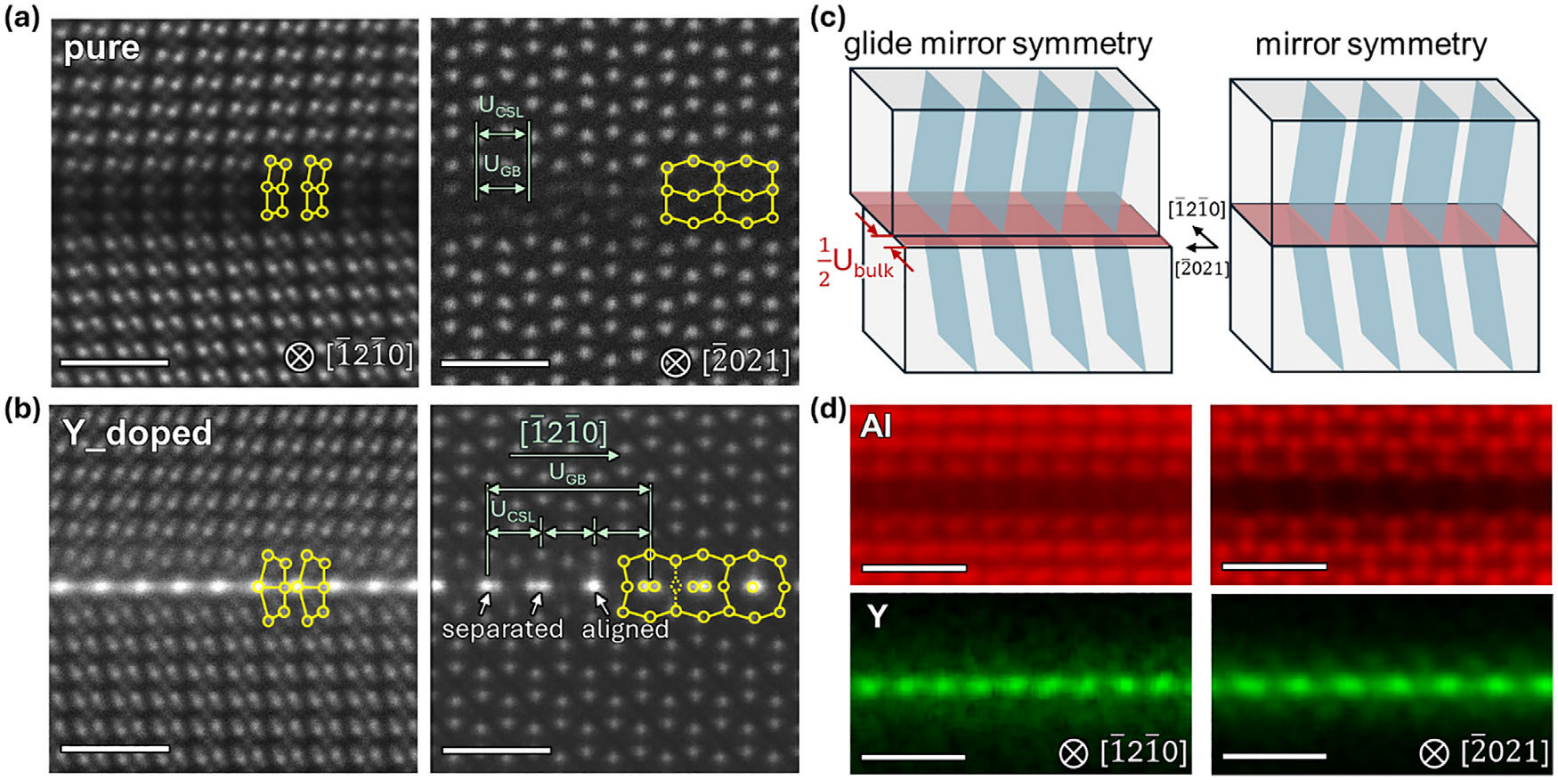
HAADF‐STEM images of a) pure ∑13(10$\bar{1}$4)/[$\bar{1}$2$\bar{1}$0] Al_2_O_3_ GB and b) Y segregated GB, both projected along the [$\bar{1}$2$\bar{1}$0] and [$\bar{2}$021] directions, with c) illustration of change in symmetry and d) the atomic‐resolved EDS results of (b). (The Y atoms show a small amount of segregation in the near‐GB atom layers, which corresponds to the minor disordering Y atoms that were observed from the planar direction before.^[^ ^25^ ^]^ Scale bar: 2 nm).

**Figure** **1** shows the experimental high‐angle annular dark‐field (HAADF)‐STEM images observed from two orthogonal directions for the pure and Y‐segregated GBs, along with the corresponding atomic resolution energy dispersive X‐ray spectrometry (EDS) maps. Since the intensity of atomic columns in a HAADF‐STEM image is positively correlated with atomic number, the brighter columns in Figure 1b can be easily recognized as Y. This is also confirmed by the EDS results in Figure 1d. As marked by the yellow dots and lines in Figure 1, the two structures show similar GB units but with different symmetries. The structure of the pure GB shown in Figure 1a has the glide mirror symmetry with respect to the middle of the two darker cation planes and is consistent with previous reports.^[^ ^23^, ^24^ ^]^ On the other hand, the Y‐segregated GB exhibits a common symmetric structure centered by the Y‐occupying layer, as illustrated in Figure 1c. Besides, the structural period of the pure GB agrees with the crystallographic prediction from the orientation relationship of the two neighboring crystals using coincidence site lattice (CSL) theory, but the Y columns in the Y‐segregated GB exhibited a separated‐separated‐aligned feature along the [$\bar{1}$2$\bar{1}$0] direction, as indicated by the white arrows in the HAADF‐STEM image taken from [$\bar{2}$021] direction in Figure 1b. This suggests that the Y‐segregated GB has a structural periodicity three times that predicted by CSL theory. Moreover, compared with the pure GB viewed from [$\bar{2}$021] direction, there seems to be a missing atom column between the two separated Y columns (as marked by a dashed circle in Figure 1b, indicating the possibility of a lower atomic density at the GB area. Therefore, the Y segregation changed not only the atomic arrangement inside the GB units but also the structure period of the GB, along with a change in GB atomic composition. This differs from the general understanding that isovalent segregation simply substitutes the host atoms and results in minor local structural adaptation without a change in GB structural period and atom density.^[^ ^11^, ^15^, ^25^ ^]^

NNP‐MCMD simulations were conducted to better understand the effect of Y atoms in modifying the GB structure. Considering the above experimental observations that the Y segregation modifies the GB structural period, we built three supercells with 1, 2, and 3 repeating units along the [$\bar{1}$2$\bar{1}$0] direction. Here, these supercells are respectively referred to as 1 × 1 × 1, 1 × 1 × 2, and 1 × 1 × 3 supercells. As demonstrated below, GB structures without a change in atom number, i.e., have the same GB atomic density as that of the pure GB, could not reproduce the experimentally observed structure. Thus, we varied atomic densities at the GB by removing a given number of Al_2_O_3_ units (one unit refers to a set of 2 Al and 3 O atoms) from the GB cell while maintaining the stoichiometry and thereby the charge neutrality.

**Figure 2:**
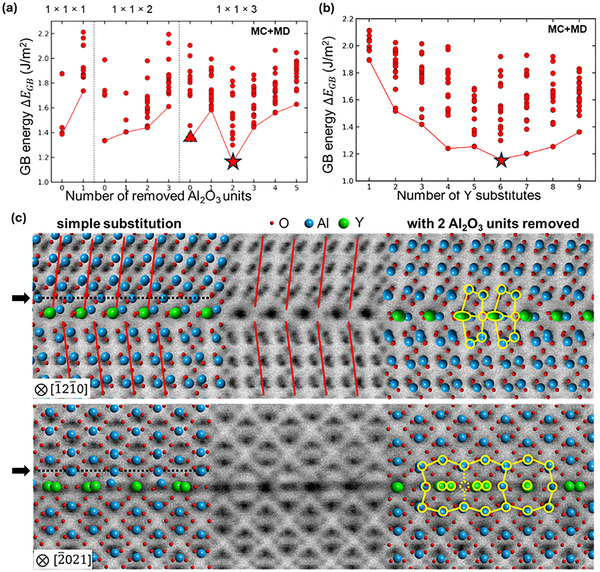
Dependence of GB energy Δ*E*_GB_ on the number of a) removed Al_2_O_3_ units and b) Y substitutes, along with c) the comparison of calculated GB structures obtained by simple substitution (marked by a triangle in (a)) and the one with the lowest Δ*E*_GB_ (marked by the star in (a) and (b)) with experimental ABF‐STEM images.

**Figure** **2**a shows the calculated Δ*E*_GB_ as a function of the number of removed Al_2_O_3_ units for the three supercells. Based on the EDS results that there are no Al signals in the Y‐occupying columns, we first introduced 2 Y, 4 Y, and 6 Y atoms (one full atom layer) into a 1 × 1 × 1, 1 × 1 × 2, and 1 × 1 × 3 supercells, respectively, by replacing Al atoms with Y atoms. It is noted that the Δ*E*_GB_ value predicted by NNP‐MCMD simulations agrees well with the corresponding density functional theory (DFT) values (see Figure S1, Supporting Information). Basically, Δ*E*_GB_ increases as removed Al_2_O_3_ units increase, but it shows a drastic drop to the lowest value of 1.7 J m^−2^ when 2 Al_2_O_3_ units are removed from the 1 × 1 × 3 supercell. To determine the Y concentration yielding the lowest‐energy state, we then varied the number of Y substitutes from 1 to 9 in the 1 × 1 × 3 supercell with the removal of 2 Al_2_O_3_ units and performed MCMD simulations on each supercell. Figure 2b displays Δ*E*_GB_ as a function of the number of Y substitutes. The Δ*E*_GB_ value initially decreases, followed by a slight increase, reaching its minimum at 6 Y substitutes. The calculated atomic structure with 6 Y substitutes is compared with the experimental annular bright‐field (ABF)‐STEM image on the right side in Figure 2c; it well reproduces the separated‐separated‐aligned feature of the center Y columns observed in STEM, and its GB units are in good agreement with the experimental images. Such a threshold is caused by the size mismatch between Y and Al, as will be discussed later.

For comparison, the atomic structure obtained by commonly used simple substitution^4^, ^11^, ^15^ with no removal of Al_2_O_3_ units (marked by a triangle in Figure 2a) is also shown on the left side in Figure 2c. As indicated with red lines, the atomic structure obtained by the simple substitution is asymmetric about the GB plane and thus is inconsistent with the symmetric arrangement of Al dumbbells observed along [$\bar{1}$2$\bar{1}$0] direction. Additionally, this structure has an extra Al layer (the black dashed line and arrows), which is absent in the experimental image. These results suggest that segregated Y atoms not only simply substitute Al atoms at the pure GB but also induce the structural modification involving changes in GB atomic density.

**Figure 3:**
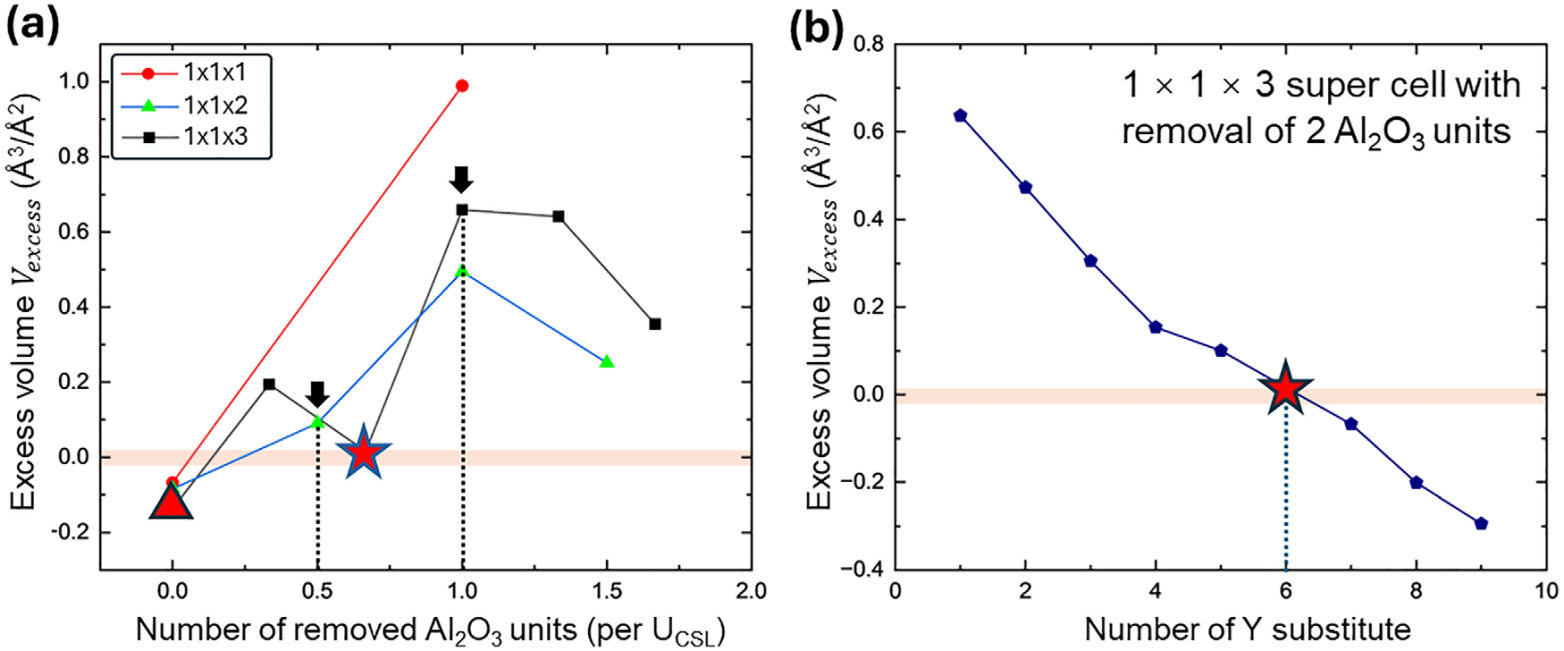
GB excess volume *V_excess_* as a function of a) the number of removed Al_2_O_3_ units per U_CSL_ and b) the number of Y substitutions in the 1 × 1 × 3 supercell with removal of 2 Al_2_O_3_ units.

To elucidate the relationship between Δ*E*_GB_ and structural features of Y‐segregated GBs, we assessed *V_excess_* for all calculated structures. Negative and positive values of *V_excess_* indicate that GB atoms have shorter and longer bond lengths than the bulk atoms on average, respectively. A near‐to‐zero *V_excess_* indicates that the bonding environment at the GB is close to that in the bulk.^[^ ^26^, ^27^, ^28^ ^]^ **Figure** **3** shows the *V_excess_* of the fully relaxed structures with different GB compositions with respect to the number of removal of Al_2_O_3_ units and Y substitutes. The number of removed Al_2_O_3_ units is normalized to each U_CSL_ to directly reflect the reduction of GB atom density. The atomic structures used in this analysis correspond to the data points on the red line in Figure 2a,b. The absolute value of *V_excess_* is found to be correlated with the magnitude of Δ*E*_GB_. More importantly, the lowest Δ*E*_GB_ structure, with 2 Y substitutes and the removal of $\frac{2}{3}$ Al_2_O_3_ units per U_CSL_ (marked by the star) has the smallest absolute value of *V_excess_*. This suggests that this GB has the bonding environment comparable to those of the Al‐O and Y‐O binary oxides (i.e., α‐Al_2_O_3_ and c‐Y_2_O_3_) and thereby the lowest‐energy state.

The overall trend of change in *V_excess_* with the GB composition can be mainly explained by the difference in bonding environment of Y^3+^ and Al^3+^, which is closely related to the ionic radii. The GB structure without removal of Al_2_O_3_ units (i.e., simple substitution of Y for Al, marked by the triangle) results in the negative *V_excess_*. This is because a Y^3+^ ion has a much larger ionic radius (0.90 Å) than that of Al^3+^ (0.54 Å),^[^ ^29^ ^]^ and would need a longer distance to form the stable Y─O bonds that are comparable to those in Y_2_O_3_. Simply substituting Al^3+^ by Y^3+^ resulted in compressed Y─O bonds similar to Al─O, with negative *V_excess_* and local stress local stress leading to the energy penalty. As the removed Al_2_O_3_ units increases, the *V_excess_* generally increases into a positive range, indicating the bonding environment is optimized and local stress is released by decreasing the atom density. However, the *V_excess_* show an abrupt drop close to zero for the lowest‐energy GB structure, indicating the emergence of some structural characteristics, which would be discussed later. This effect of ion radii on *V_excess_* is further confirmed by modifying the number of Y substitutes in the stable structure with the closest‐to‐zero *V_excess_*, as shown in Figure 3b. For the removal of 2 Al_2_O_3_ units in the 1 × 1 × 3 supercells, an increasing number of Y substitutes monotonically decreases the *V_excess_*, indicating transition from an over‐relaxed bonding environment to an over‐compressed bonding environment.

**Figure 4:**
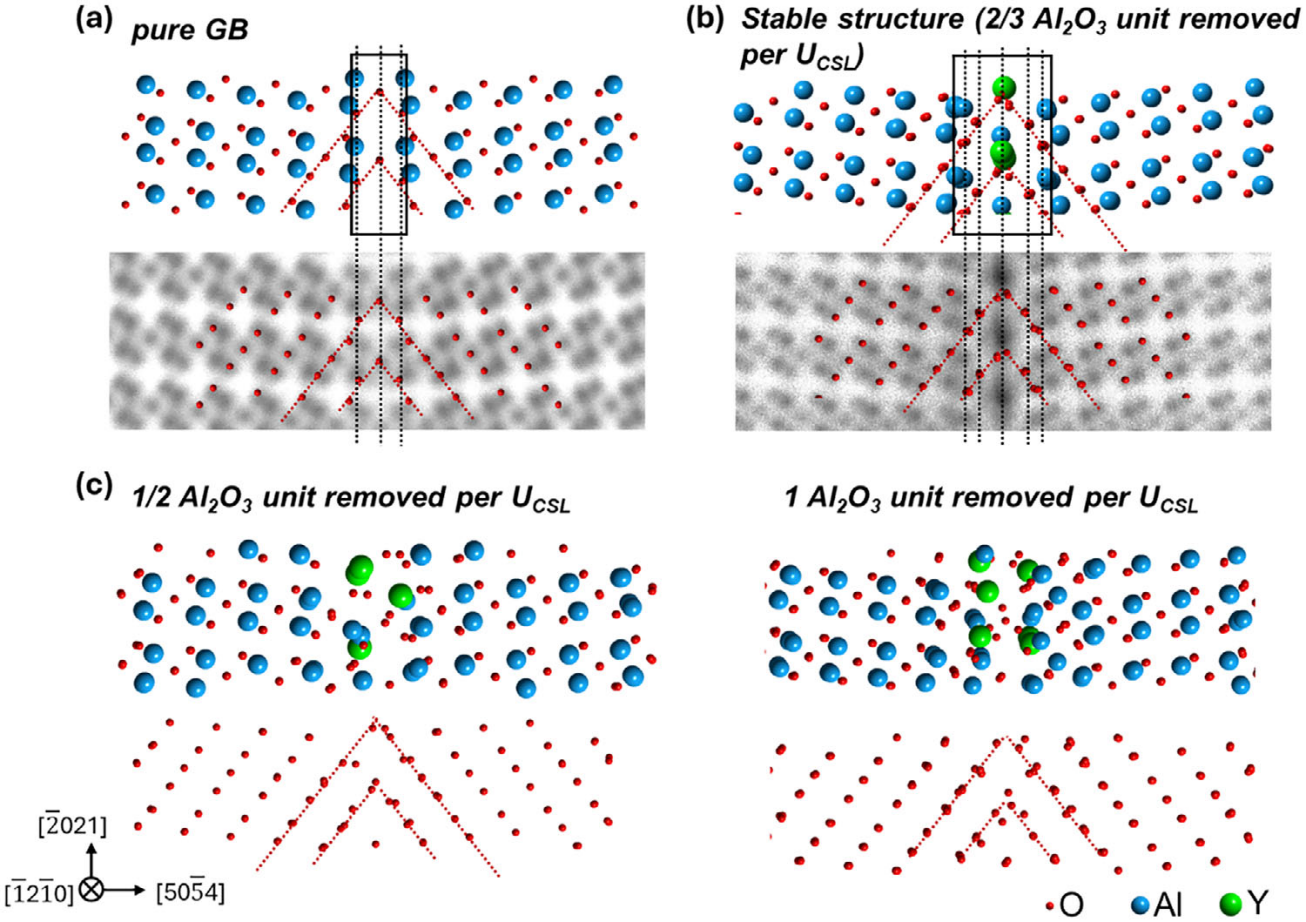
Structural comparison of the a) pure and b) stable structure (with 2/3 Al_2_O_3_ unit removed per U_CSL_) with c) the representative structures with a random O sublattice and large *V_excess_* (with 1/2 and 1 Al_2_O_3_ unit removed per U_CSL_, corresponding to dots marked with black arrows in Figure 3a).

To reveal the structural characteristic that resulted in the abrupt drop of *V_excess_* of the most stable structure (marked by the star in Figure 3a), we further compared the atomic structures of all calculated Y‐segregated GBs, including those unfavored ones, along with the pure GB. The atomic structure of the pure GB is shown in **Figure** **4**a. It is a compact GB with the two crystals relatively rotated by 180° about the [50$\bar{5}$4] axis. Due to this symmetry, the (10$\bar{1}$4) O planes retain their closely‐packed configuration across the GB, forming a compact O sublattice (discussed in detail in Note S1 and Figure S2, Supporting Information). The most stable Y‐segregated GB structure is shown in Figure 4b, and representative higher‐energy structures obtained from MCMD simulations are shown in Figure 4c. A comparison of the four structures in Figure 4 reveals that the Y‐segregated structure in Figure 4b exhibits an O sublattice highly similar to that of the pure GB. Their O sublattices have the same symmetric arrangement, with the same atom density at all columns, thereby retaining the compactness of the near hexagonal close‐packed O sublattice in α‐Al_2_O_3_. Considering that O^2−^ has a larger ionic radius (1.40 Å) than Al^3+^ (0.54 Å) and Y^3+^ (0.90 Å), preserving the compactness of the O sublattice could greatly contribute to reducing the *V_excess_* and thereby Δ*E*_GB_. This interpretation is supported by the fact that the pure and lowest‐energy Y‐segregated GBs have near‐to‐zero *V_excess_* values of ≈0.032 Å^3^/Å^2^ and ≈0.0158 Å^3^/Å,^2^ respectively. On the other hand, the O positions of the higher‐energy structures exhibit larger deviations from the compact O sublattice indicated by the red dashed lines, as exhibited in Figure 4c. (Figure 4c shows representative examples with removing 1/2 and 1 Al_2_O_3_ unit per U_CSL_, corresponding to black arrows in Figure 3a, and all the other structures are exhibited in Figure S3, Supporting Information). Such structure characteristics result in *V_excess_* larger than that of the lowest‐energy structure in Figure 4b. Therefore, the abrupt drop of *V_excess_* at 2/3 Al_2_O_3_ unit removed per U_CSL_ can be attributed to the reformation of the compact O sublattice.

In addition, the emergence of the threefold repeat structure is also closely related to the characteristic compact O sublattice. In a compact O sublattice in the 1 × 1 × 1 supercell, each (10$\bar{1}$4) plane has two O^2−^. While the ‐Al‐Al‐O‐O‐O‐ sequence is well preserved in the pure GB for the 1 × 1 × 1 supercell and thus maintains the charge neutrality,^[^ ^30^ ^]^ as marked by a rectangle in Figure 4a, the Y‐segregated GB structure has 5 O planes (10 O^2−^) between the bulk Al planes (10$\bar{1}$4) (as marked by rectangle and dashed lines in Figure 4b) in a 1 × 1 × 1 supercell, and needs extra $\frac{8}{3}$ M^3+^ to maintain the charge neutrality (except for the 4 Al^3+^ in the nearest Al dumbbells included in the rectangle). Therefore, a triple period is necessary to ensure an integer number of cations and to stabilize such GB configurations.

With this knowledge, it is concluded that the Y segregation process could not occur by simple substitution without changes in atomic density at the GB, as this results in a large negative *V_excess_* along with compressed bonding environment and local stress, and hence makes the GB structure energetically unfavorable. Instead, a modification in the GB atomic composition through decreasing the atomic density can optimize the local bonding environment, resulting in near‐zero *V_excess_* and thereby the lowest Δ*E*_GB_. Such an effect is well supported by our NNP‐based MCMD calculations for a 1 × 1 × 3 ∑13 GB supercell with the removal of 2 Al_2_O_3_ units, which well reproduced the experimental images. This highlights that isovalent segregation processes can be a more complex phenomenon than simple substitution, potentially involving point defect emission and absorption.^[^ ^22^ ^]^

## 3 Conclusion

In conclusion, our study demonstrated that Y segregation at the ∑13(10$\bar{1}$4)/[$\bar{1}$2$\bar{1}$0] GB in α‐Al_2_O_3_ involves not only a simple substitution process but also structural adaptation associated with a change in atomic density at the GB. The optimized bonding situation, which should have all mitigated strain fields, well‐balanced overall and local charge neutralization, is the critical factor in determining the preferred GB composition as well as the atomic structure. STEM observations and NNP‐MCMD simulations revealed that a certain Y concentration and atomic density result in a near‐zero *V_excess_* with the atomic bonding environment close to those in α‐Al_2_O_3_ and c‐Y_2_O_3_, leading to the formation of the lowest‐energy structure. This insight provides an atomic‐scale understanding of how dopant segregation alters GB structure, highlighting the broader connection between GB crystallography, defect chemistry, and dopant characteristics. Our findings offer a new perspective on the impurity‐GB interaction, which is essential for interpreting the defect behavior and guiding future studies on ceramic microstructure engineering.

## 4 Experimental Section

### Sample Preparation

In this study, a bicrystal sample was used to obtain a well‐defined model GB.^[^ ^31^ ^]^ The bicrystallographic orientation is summarized as (10$\bar{1}$4)_top_//(10$\bar{1}$4)_bottom_, [1$\bar{2}$10]_top_//[$\bar{1}$2$\bar{1}$0]_bottom_ and [$\bar{2}$021]_top_//[20$\bar{2}\bar{1}$]_bottom_. Y segregation was introduced by a precise etching/coating system (PECS, Model 685, Gatan Inc.) before bonding. A broad argon ion beam was illuminated on the pure Y metal target, and the sputtered Y atoms were then deposited on the surface of the single crystal (Shinkosha Co., Ltd.) placed under the target. In the meantime, the thickness of the coated thin film was monitored by a thickness sensor, and the coating process would be stopped once the thickness reaches the set value. In this research, a 3 nm‐ thick Y film was coated. The coated single crystals were bonded by thermal diffusion bonding at 1500 °C under 0.1 MPa for 30 h in air.^[^ ^24^ ^]^

TEM samples with the well‐defined GBs placed in the middle were prepared by focused ion beam (FIB) (Helios 5, FEI). TEM lamellas sized 6 µm × 8 µm × 1 µm containing a single GB were extracted from the bulk bicrystal. The lamellas were welded to copper mesh and further thinned to ≈80 nm according to the parameters suggested in the previous report.^[^ ^32^ ^]^

### STEM Characterization

The STEM characterizations were conducted using an ARM‐200CF (JEOL Ltd.) equipped with an aberration corrector and a cold field emission gun operated at 200 kV. The convergence semi‐angle was 23.5 mrad, and the collection semi‐angle was 68–280 mrad for high‐angle annular dark‐field (HAADF) and 12–24 mrad for annular bright‐field (ABF) imaging.

STEM‐Energy Dispersive X‐ray Spectrometry (EDS) characterization was conducted. STEM–EDS mappings were acquired by scanning the beam near the GB, using the NSS3 software developed by Thermo Fisher Scientific Inc. The STEM–EDS system was equipped with double SDD‐EDS detectors, and the solid angle for the whole collection system was ≈1.7 sr. The probe size was 1.2 Å with a probe current of ≈60 pA. EDS spectra were extracted with selected EDS energy for each element: Al (Kα of 1.486 keV), O (Kα of 0.525 keV) Y (Kα of 14.931 keV and Kβ of 16.731 keV).

### MCMD Simulation Based on Neural Network Potential

Monte Carlo (MC) and molecular dynamics (MD) simulations (hereafter referred to as MCMD simulations) were performed to predict the lowest‐energy atomic structure of the Y segregated GB, as mentioned below. A neural network potential (NNP) was implemented to calculate the potential energy and the force acting on each atom in MCMD simulations. The NNP was demonstrated to accurately predict the energetics of both pure and Y‐segregated Al_2_O_3_, as provided in the Supporting Information (Notes S2 and S3, Figures S4–S6, Supporting Information).

In an MCMD simulation, an initial structure was built from a pure GB cell by randomly replacing Al with Y atoms. The predicted pure GB was found to have the same atomic structure as that in a previous DFT study.^[^ ^23^ ^]^ An MC simulation was first performed, during which the occupation site of an Al atom was exchanged with that of a Y atom by randomly selecting one Y atom within 4 Å of the Al atom. The acceptance rate *p* for the exchange operation was given by:

(1) $$p=\left\{\begin{array}{c} 1\;\;\;\;\;\;\;\;\;\;\;\;\;\left(E_{\mathrm{after}}-E_{\mathrm{before}}\leq 0\right)\;\\ \exp\left(-\frac{E_{\mathrm{after}}-E_{\mathrm{before}}}{k_{B}T}\right)\;\left(E_{\mathrm{after}}-E_{\mathrm{before}}>0\right)\; \end{array}\right.$$

where *E*_before_ and *E*_after_ are the total potential energies before and after an exchange, which were obtained after structural relaxation, and *k*_B_ is the Boltzmann constant. The MC temperature *T* was set to 1000 K. The number of trial exchanges was set to 500 in one MC period. An MD simulation was then performed for 100 ps with a timestep of 1 fs at 2000 K to obtain a snapshot every 5 ps. The reasons for the temperature selection are illustrated in Note S4 (Supporting Information). The obtained snapshots were fully relaxed with structural optimization, and the lowest‐energy one was used as an initial cell in a subsequent MC simulation based on the Metropolis algorithm. A set of the MD and MC steps was carried out 10 times. The MCMD method provides sufficient convergence, as discussed in detail in Note S5 and Figures S7 and S8 (Supporting Information).

Note that final atomic structures possibly depend on initial Y configurations, randomness of exchange operation in MD simulations, and initial velocity distributions in MD simulations. Thus, 10 different Y configurations were initially generated to perform 10 independent MCMD simulations for a given number of Y atoms. In addition, two GBs were present in one supercell due to 3D periodic boundary conditions and were treated independently in MCMD simulations, and thus 20 GB structures were obtained for one Y concentration. The lowest‐energy one was finally identified by comparing its GB energies Δ*E*_GB_ in the form:

(2) $$\Delta E_{\mathrm{GB}}=\frac{E_{\mathrm{GB}}\left(mAl_{2}O_{3},nY_{2}O_{3}\right)-mE_{\mathrm{BULK}}^{Al_{2}O_{3}}-nE_{\mathrm{BULK}}^{Y_{2}O_{3}}}{2A}\;$$

where *E*_GB_(*m*Al_2_O_3_,*n*Y_2_O_3_) is the potential energy of a GB cell containing *m*Al_2_O_3_ and *n*Y_2_O_3_ units, *A* is the area of the GB plane, and $E_{\mathrm{BULK}}^{Al_{2}O_{3}}$ and $E_{\mathrm{BULK}}^{Y_{2}O_{3}}$ are the potential energies per Al_2_O_3_ and Y_2_O_3_ unit for the perfect lattices, respectively. The values of $E_{\mathrm{BULK}}^{Al_{2}O_{3}}$ and $E_{\mathrm{BULK}}^{Y_{2}O_{3}}$ were respectively obtained from pure α‐Al_2_O_3_ and c‐Y_2_O_3_. To evaluate the predictive ability of the NNP on Δ*E*_GB_, DFT single‐point calculations were performed using the Y‐segregated GB structures obtained from NNP‐based MCMD simulations, as will be shown in the Supporting Information.

### Calculation of the Overall GB Excess Volume

The atomic excess volume of the i‐th atom *V*_*excess* − *i*_ was defined by:

(3) $$V_{excess-i}=V_{GB-i}-V_{bulk-i}$$

where *V*_*GB* − *i*_ is the Voronoi volume of the i‐th atom at a GB, and *V*_*bulk* − *i*_ is the mean Voronoi volume in the perfect lattice. The averaged Voronoi volumes in α‐Al_2_O_3_ and c‐Y_2_O_3_ were taken as *V*_*bulk* − *i*_ for Al and Y atoms, respectively. Therefore, the overall GB excess volume could also be expressed by:

(4) $$V_{excess}=\frac{V\left(mAl_{2}O_{3},nY_{2}O_{3}\right)-mV_{\mathrm{BULK}}^{Al_{2}O_{3}}-nV_{\mathrm{BULK}}^{Y_{2}O_{3}}}{2A}$$

where $V_{\mathrm{BULK}}^{Al_{2}O_{3}}$ and $V_{\mathrm{BULK}}^{Y_{2}O_{3}}$ are the Voronoi volumes of one unit of α‐Al_2_O_3_ and c‐Y_2_O_3_ in the single crystals.

## Conflict of Interest

The authors declare no conflict of interest.

## Supporting information



Supporting Information

## Data Availability

The data that support the findings of this study are available from the corresponding author upon reasonable request.
