# Supplementary material for: Atomic‐Scale Insights into Yttrium‐Induced Grain Boundary Structure Modification in Al2O3
Source: Adv Sci (Weinh). 2025 Dec 22;13(17):e15350. doi: 10.1002/advs.202515350 (PMC13042377; doi:10.1002/advs.202515350)
Supplement: Supplementary file 1 — Supporting Information [file ADVS-13-e15350-s001.docx]

Supporting Information

**Atomic-scale Insights into Yttrium-Induced Grain Boundary Structure Modification in Al_2_O_3_**

*Jingyuan Yan, Tatsuya Yokoi, Yuuki Nakano, Shun Kondo*, Bin Feng, Naoya Shibata, Katsuyuki Matsunaga & Yuichi Ikuhara**

**Figure S1-S8**

Table S1-S2

Note S1-S5

Reference 33-47

**Figure S1. Comparison of the GB energy calculation results of the NPP-based MCMD with the traditional DFT method.** (a) $\Delta E_{\mathrm{GB}}$ of structures with different super cells as a function of different number of Al_2_O_3_ units removed (6 Y substitutes). (b) The correlation of $\Delta E_{\mathrm{GB}}$ and number of U substitutes in the 1 × 1× 3 supercell with 2 Al_2_O_3_ units removed. The $\Delta E_{\mathrm{GB}}$ value predicted by NNP-based MCMD simulations (upper panel) agrees quantitatively with the corresponding DFT values (lower panel). Most importantly, the red star in Fig. 2(a) indicates that the lowest-energy structure for the NNP is the same as that for DFT calculations

**
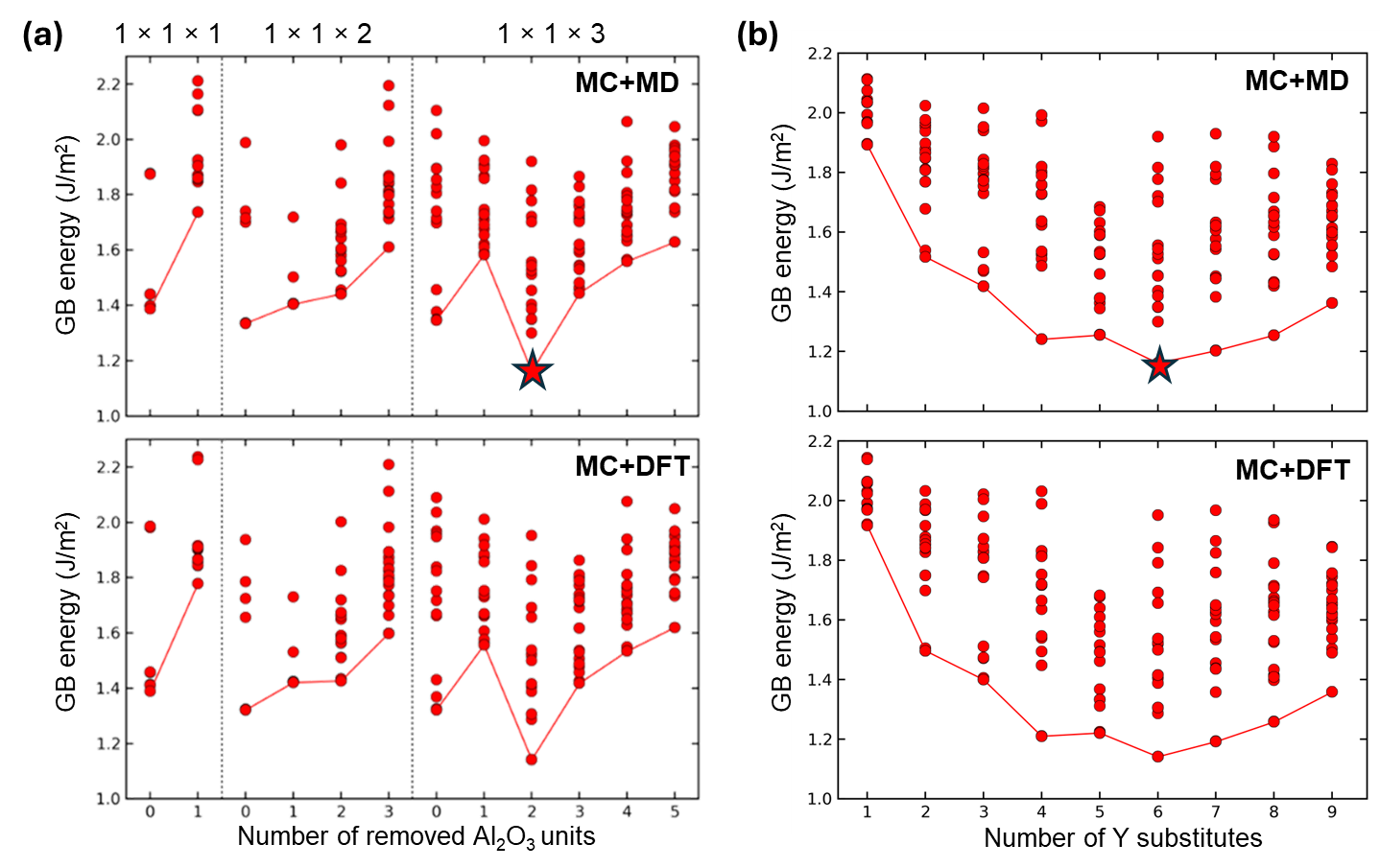
**

**Figure S2. Illustration of the compactness of O sublattice in pure ∑13(10**$\bar{\boldsymbol{1}}$**4)/[**$\bar{\boldsymbol{1}}$**2**$\bar{\boldsymbol{1}}$**0] Al_2_O_3_ GB.** (a) Schematic of the three-dimensional stacking sequence; (b) The plane view of the upper (U1-U3) and lower (L1-L3) O (10$\bar{1}$4) planes with hexagon repeating units.


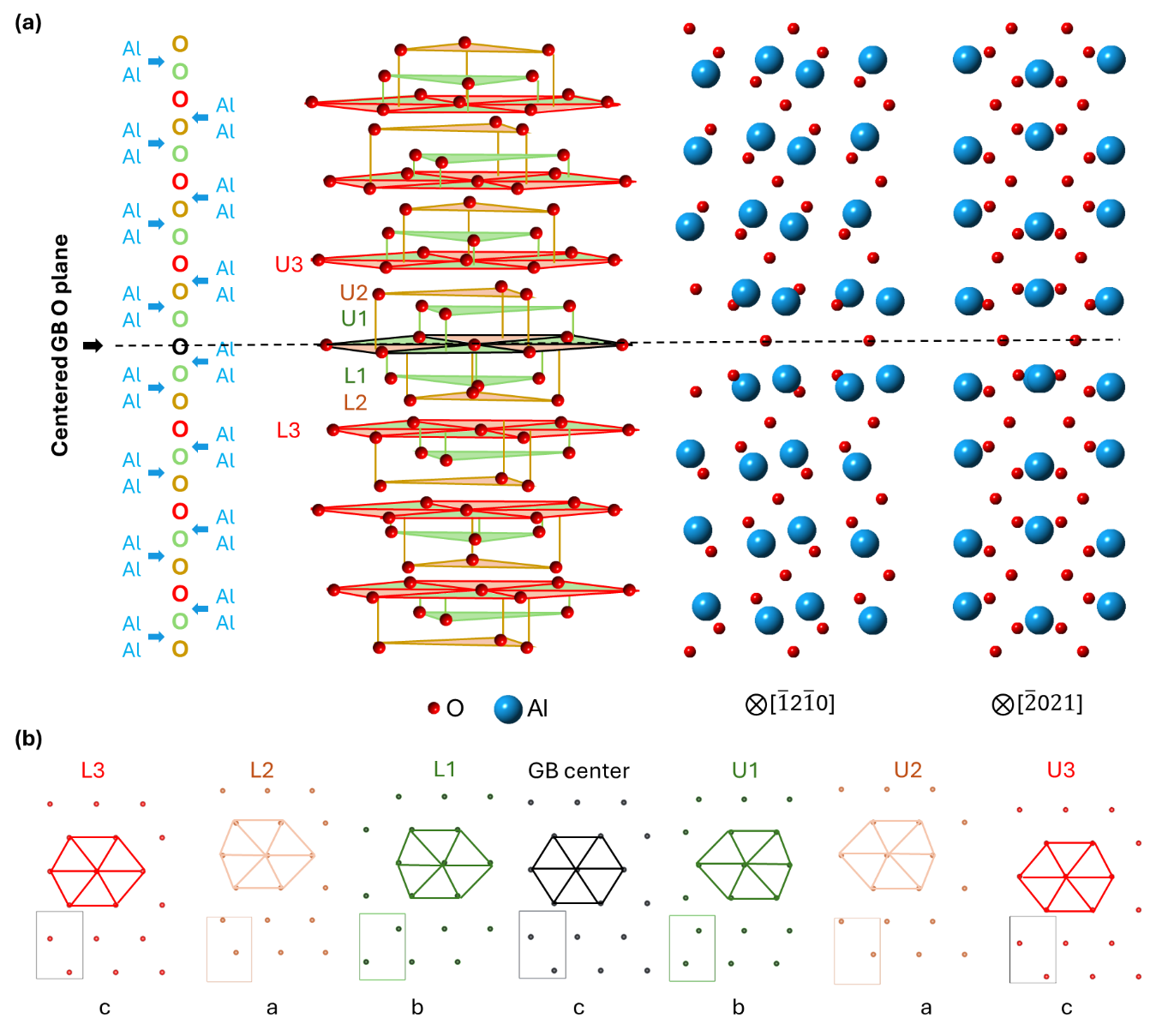


**Figure S3. All atomic structures obtained by the NNP-based MCMD calculations with different removal of Al_2_O_3_ units in 1 × 1 × 1, 1 × 1 × 2 and 1 × 1 × 3 superlattices.**

**
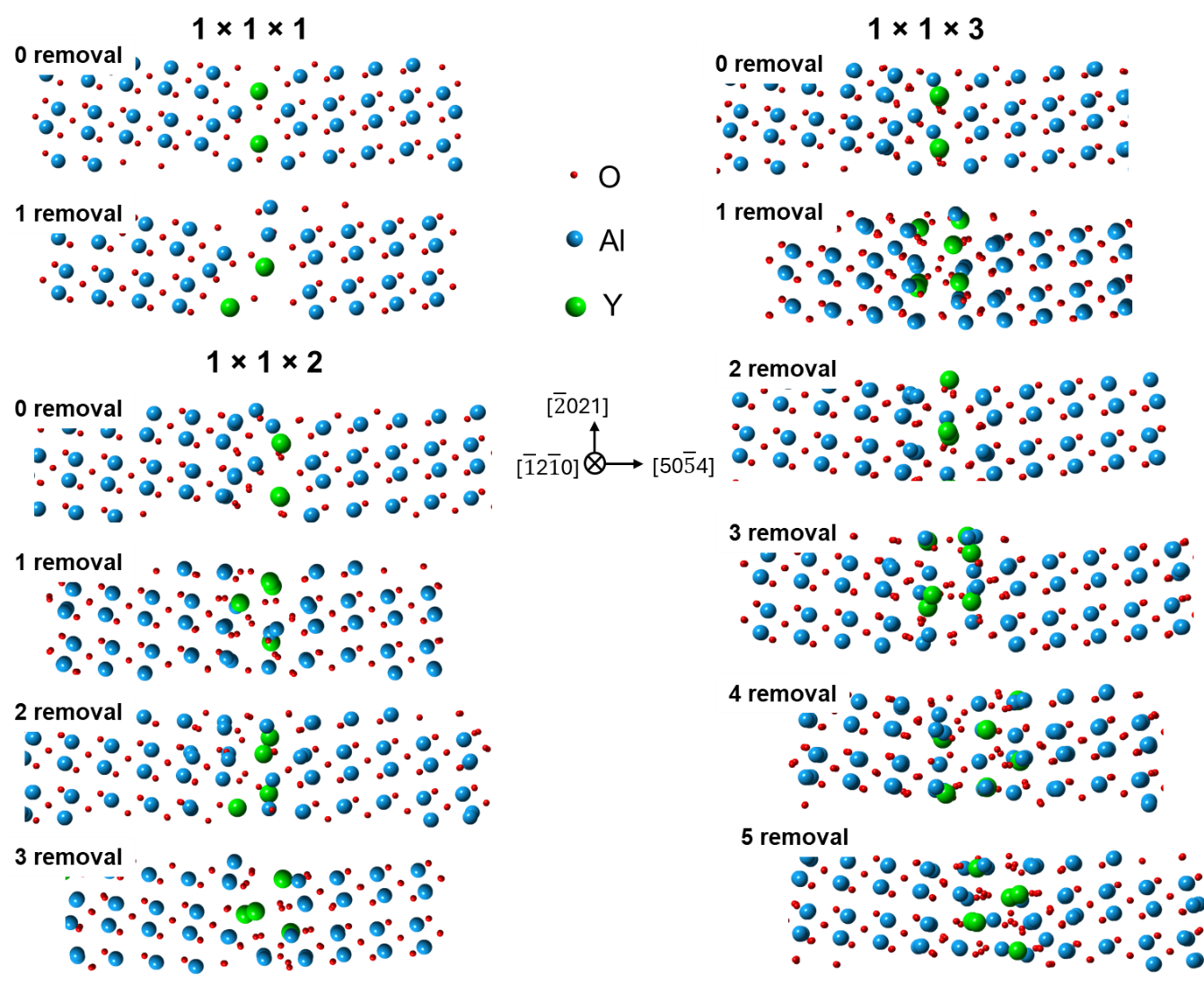
**

**Figure S4. Error of the NNP in MD simulations for (a) the pristine bulk lattice and (b) the pristine** $\boldsymbol{\Sigma}\boldsymbol{13}\boldsymbol{(}\boldsymbol{10}\bar{\boldsymbol{1}}\boldsymbol{4}\boldsymbol{)}$ **GB. (c) and (d) Mean absolute errors (MAE) for the potential energy and atomic force, respectively.** A 2 × 2 × 1 supercell of a conventional corundum unit cell for α-Al_2_O_3_ was used for the bulk lattice, containing 48 Al and 72 O atoms. For the $\Sigma13$ GB, a supercell with 64 Al and 96 O atoms was constructed in three-dimensional periodic boundary conditions. The distance of two GB planes was set to 21.2 Å. An NNP-MD simulation was initially performed for 30 ps at each of the temperatures, and an MD snapshot was extracted at every 10 ps. The obtained snapshots were used to perform DFT single-point calculations.


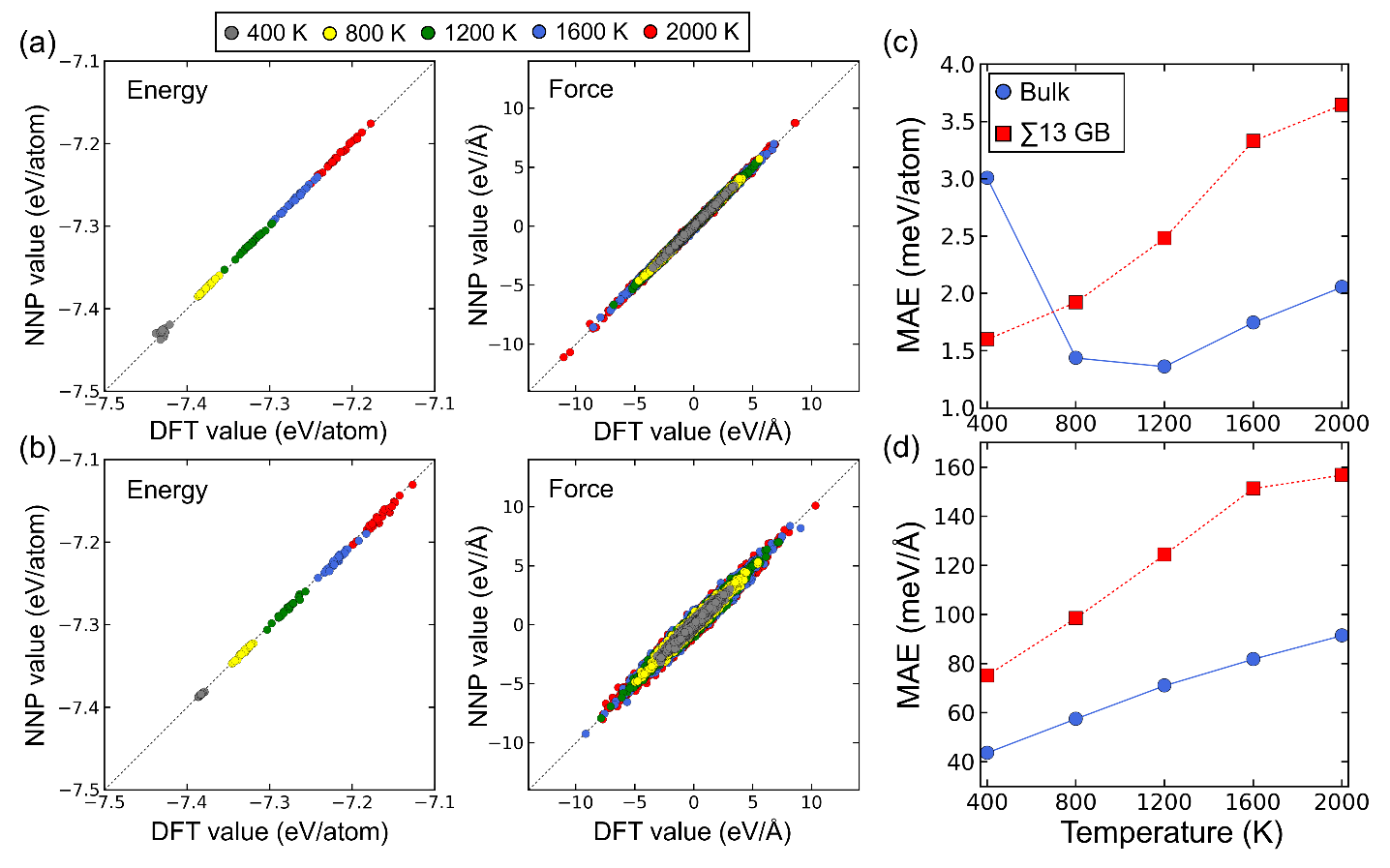


**Figure S5. Error of the NNP in MD simulations for (a) the Y-segregated bulk lattice and (b) the Y-segregated** $\boldsymbol{\Sigma}\boldsymbol{13}$ **GB. Three Y concentrations were examined for the two systems, as indicated at the bottom right of each of the panels. (c) and (d) Mean absolute errors (MAE) for the potential energy and atomic force, respectively.** For a given Y concentration and temperature, an initial structure was built by randomly substituting Y for Al atoms. The other computational conditions are the same as those of the pristine system as described in Fig. S4.


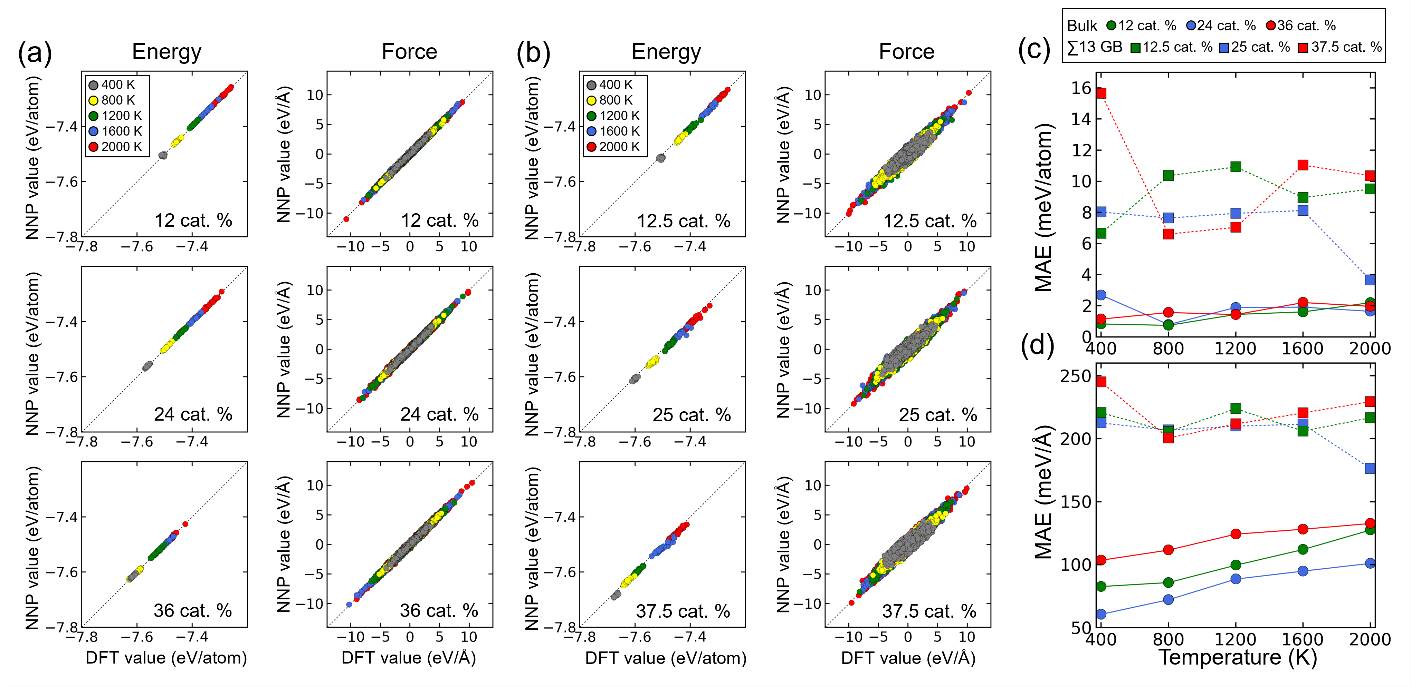


**Figure S6. Error of the NNP for the Y-segregated GB structures obtained from MCMD simulations. The GB energies were calculated by Eq. (1) in Experimental section in main text.**


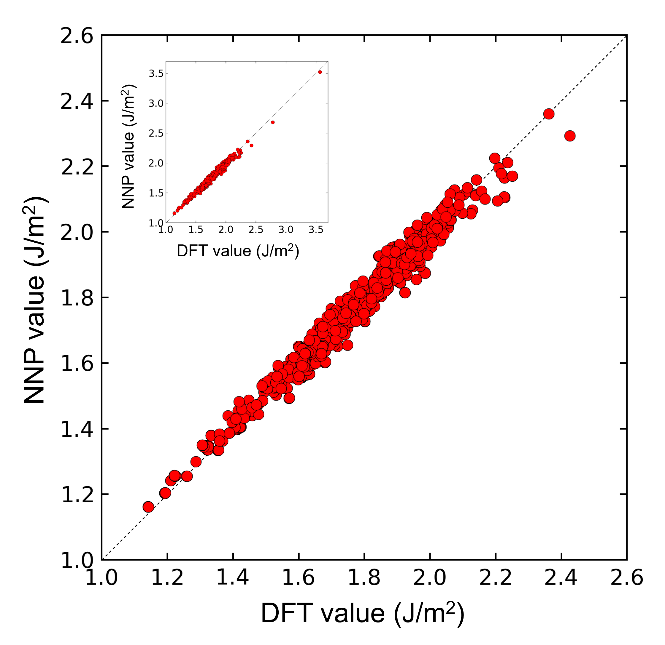


**Figure S7. Energy decrease as a function of the number of trial moves in MC simulations for 10 independent MCMD simulations for the 1 × 1 × 3 supercell with 2 removed Al_2_O_3_ units and 6 Y atoms. The vertical lines indicate an MD simulation between successive MC simulations.**


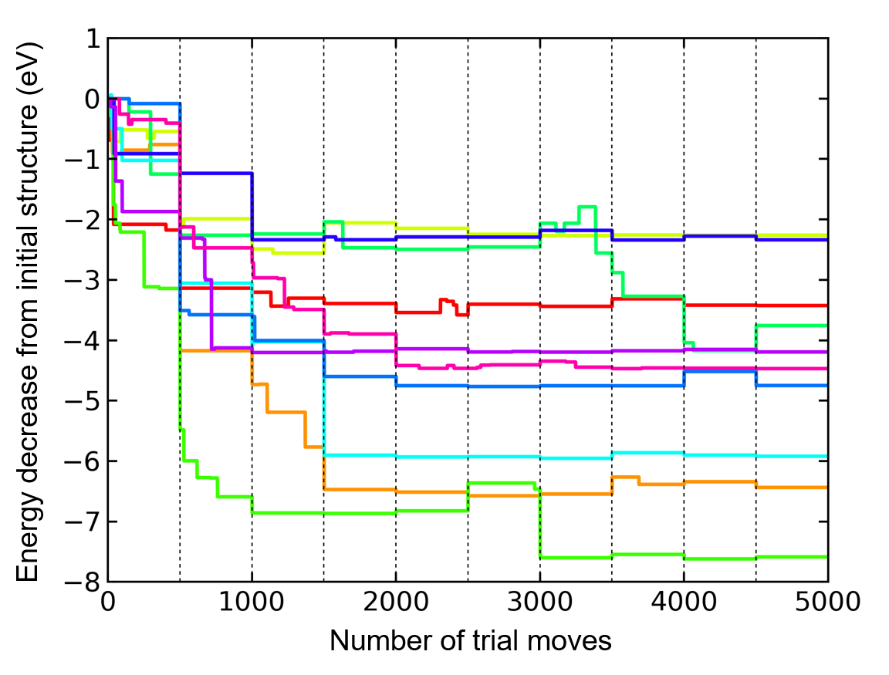


**Figure S8. Procedure of reconstructing two supercells from the simulation cell obtained from an MCMD simulation.**


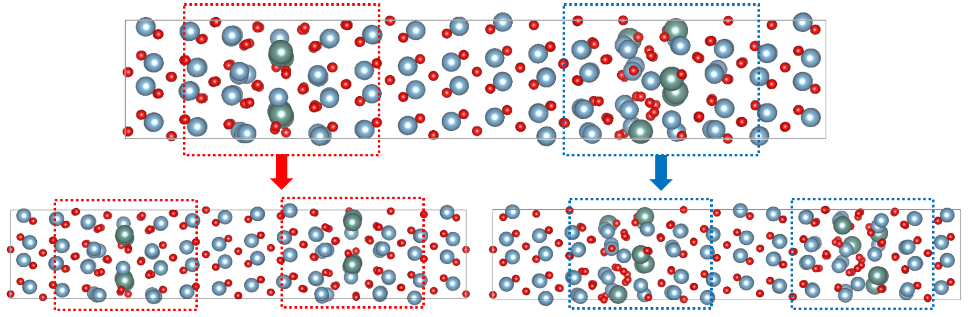


**Table S1. Training datasets for the NNP**

| Reference structure | | Amount of data | | |
| --- | --- | --- | --- | --- |
|  |  | Energy | Force | Stress |
| Bulk lattice | 1 × 1 × 1 cell | 18850 | 1661625 | 113100 |
|  | 2 × 2 × 1 cell | 2187 | 741960 | 13122 |
| Surface | | 5325 | 798750 | 31950 |
| STGB with  $[\bar{1}2\bar{1}0]$ axis | $\Sigma37(10\bar{1}\bar{8})$ | 4775 | 1582500 | 28650 |
|  | $\Sigma11(10\bar{1}1)$ | 5775 | 1116000 | 34650 |
|  | $\Sigma3(10\bar{1}0)$ | 4875 | 967500 | 29250 |
|  | $\Sigma3(0001)$ | 4875 | 960000 | 29250 |
|  | $\Sigma13(10\bar{1}4)$ | 4875 | 645000 | 29250 |
|  | $\Sigma7(10\bar{1}\bar{2})$ | 4875 | 645000 | 29250 |
|  | $\Sigma7(10\bar{1}2)$ | 4775 | 1891500 | 28650 |
| Y_3_Al_5_O_12_ (YAG) | | 1300 | 624000 | 7800 |
| Y_4_Al_2_O_9_ (YAM) | | 1900 | 342000 | 11400 |
| YAlO_3_ (YAP) | | 1900 | 114000 | 11400 |
| c-Y_2_O_3_ | | 798 | 191520 | 4788 |
| Total | | 67085 | 12281355 | 402510 |

**Table S2. Lattice constants predicted by the NNP and DFT calculations**

| Phase | Lattice constant | |
| --- | --- | --- |
|  | NNP | DFT |
| α-Al_2_O_3_ | $a=b=4.807 Å, c=13.129 Å$  $\alpha=\beta=90^{\circ},\gamma=120^{\circ}$ | $a=b=4.807 Å, c=13.115 Å$  $\alpha=\beta=90^{\circ},\gamma=120^{\circ}$ |
| YAG | $a=b=c=12.109 Å$  $\alpha=\beta=\gamma=90^{\circ}$ | $a=b=c=10.103 Å$  $\alpha=\beta=\gamma=90^{\circ}$ |
| YAM | $a=7.407 Å,b=10.529 Å, c=11.237 Å$  $\alpha=90^{\circ},\beta=108.39^{\circ},\gamma=90^{\circ}$ | $a=7.408 Å,b=10.526 Å, c=11.222 Å$  $\alpha=90^{\circ},\beta=108.45^{\circ},\gamma=90^{\circ}$ |
| YAP | $a=5.392 Å,b=7.436 Å, c=5.229 Å$  $\alpha=\beta=\gamma=90^{\circ}$ | $a=5.380 Å,b=7.437 Å, c=5.213 Å$  $\alpha=\beta=\gamma=90^{\circ}$ |
| c-Y_2_O_3_ | $a=b=c=10.675 Å$  $\alpha=\beta=\gamma=90^{\circ}$ | $a=b=c=10.653 Å$  $\alpha=\beta=\gamma=90^{\circ}$ |

**Note S1: Compactness of O sublattice in the pure and Y-segregated** $\boldsymbol{\Sigma13}\mathbf{(}\mathbf{10}\bar{\mathbf{1}}\mathbf{4}\mathbf{)/[}\bar{\mathbf{1}}\mathbf{2}\bar{\mathbf{1}}\mathbf{0}\mathbf{]}$ **GB**

The bulk Al_2_O_3_ lattice is characterized by the nearly hexagonal-close-packed O sublattice and Al atoms residing in 2/3 octahedral interstitials. When viewed from the [$\overline{1}$2$\overline{1}$0] direction, two (10$\bar{1}$4) Al planes reside consecutively every 3 O planes, forming a -O-O-O-Al-Al- stacking sequence along the [$50\overline{5}$4] direction. The O atoms in each plane have a hexagon repeating unit, and the O planes follow an -a-b-c-a-b-c- stacking sequence that similar to the stacking rule of HCP lattices. The three-dimensional stacking sequence of the pure $\Sigma13(10\bar{1}4)/[\bar{1}2\bar{1}0]$ Al_2_O_3_ GB is visualized in Figure S3(a). As for the ∑13 Al_2_O_3_ GB, the two crystals are relatively rotated by 180° along [$50\overline{5}$4] direction, and the (10$\bar{1}$4) O planes follows the -a-b-c-b-a- stacking sequence and therefore could keep the compactness of O as shown in Figure S2(b).

**Note S2:** **DFT and training condition of neural-network potential**

DFT calculations were performed by employing the Vienna Ab initio Simulation Package (VASP) ^[33,34]^ with the projector augmented wave (PAW) approach.^[35,36]^ PAW pseudopotentials were used for valence electron configurations of 3*s*^2^3*p*^1^, 4*s*^2^5*s*^1^4*p*^6^4*d*^2^ and 2*s*^2^2*p*^4^ for Al, Y, O, respectively. The generalized gradient approximation based on the Perdew-Burke-Ernzerhof (GGA-PBE) functional^[37]^ was used to calculate the exchange-correlation energy. The energy cutoff for plane waves was set to 500 eV. The convergence criterion of total energy was set to 10^-6^ eV. In *k*-point sampling, a Γ-centered 6 × 6 × 2 mesh was used for a conventional unit cell of α-Al_2_O_3_. DFT-MD simulations based on the Parrinello-Rahman dynamics^[38,39]^ were performed with a timestep of 2 fs.

A neural-network potential (NNP) with two hidden layers consisting of 40 nodes was implemented on the basis of the architecture in the literature.^[40-42]^ The hyperbolic tangent was chosen to be an activation function. A crystal structure was encoded using a descriptor based on the Chebyshev polynomials^[43]^ with a cutoff radius of 5 Å. This value was empirically determined in our previous studies.^[44,45]^ A cutoff function based on the hyperbolic tangent^[42]^ was used to ensure that interatomic interaction smoothly decays with increasing interatomic distance and vanishes at 5 Å. The descriptor was assumed to have 24 radial and 96 angular terms to describe the atomic environment of each atom. Note that the original paper of this descriptor^[43]^ introduced weight parameters differing with chemical species to describe the chemical environment surrounding an atom. This can avoid separating different combinations of pairs and triples of chemical species, so that the complexity of the descriptor is constant regardless of the number of chemical species. However, this study adopted the original implementation of NNPs^[40-42]^ for which different pairs and triples of chemical species are assigned to different nodes at the input layer. In our experience, this original implementation has a higher resolution of chemical environment in space and thereby smaller errors than that with weight parameters mentioned, although its complexity grows quadratically with the number of chemical species. An extended Kalman filter algorithm^[46,47]^ was used for training the NNP.

The training datasets are summarized in Table S1. Not only the bulk lattice but also defected lattices involving native point defects, surfaces and symmetric tilt GBs (STGB) were used as reference structures in order for the datasets to cover a wide variety of atomic configurations. Both pristine and Y-segregated systems were considered by randomly substituting Y for Al. Y_3_Al_5_O_12_ (YAG), Y_4_Al_2_O_9_ (YAM), YAlO_3_ (YAP) and c-Y_2_O_3_ were also considered as they are the major phases for the Al-Y-O ternary system. The training datasets were built from these reference structures by performing structural relaxation and MD simulation in the following way. For structural relaxation, an initial structure was generated by displacing randomly-selected atoms and by distorting the supercell. The structure was then relaxed by performing DFT calculations for ~10 iterations, and snapshots were added to the datasets. DFT-MD simulations were also performed at 200-2800 K to add MD snapshots to the datasets. Furthermore, the NNP trained on the above DFT data was used to generate additional datasets that specifically sample near-equilibrium atomic environments by performing NNP-based structural relaxation and MD simulation. For structural relaxation, an initial structure was generated as mentioned above and was relaxed with the NNP. The fully relaxed structure was used to perform a DFT single-point calculation and to add its DFT data to the datasets. MD snapshots were also obtained by performing NNP-MD simulations for several hundreds of picoseconds and were added to the datasets. Finally, the NNP was again trained on all training datasets mentioned.

The mean absolute errors (MAE) for all training datasets are evaluated to be 3.7 meV/atom, 125.3 meV/Å and 4.5 kbar for the energy, force and stress, respectively. These values for the energy and force are comparable to our previous NNP, which demonstrated the ability to accurately predict the site preference of Y atoms at GBs in α-Al_2_O_3_.^[44]^ Table S2 shows that the trained NNP well reproduces the DFT lattice constants of the major phases for the Al-Y-O system, within errors of 0.022 Å and 0.06$^{\circ}$ in dimension and angle, respectively.

**Note S3: Predictive ability of neural-network potential**

This section examines whether the NNP predicts accurately the energetics for pristine and Y-segregated Al_2_O_3_, which is essential to perform NNP-based Monte Carlo (MC) and MD (MCMD) simulations with satisfactory accuracy. Figure S4 shows errors in MD simulations for the pristine bulk lattice and $\Sigma13(10\bar{1}4)/[\bar{1}2\bar{1}0]$ GB. The NNP is found to accurately predict the potential energy and force acting on each atom for both systems, as data points are distributed near the diagonal line without significant deviation. As indicated by Figure S3(c) and S3(d), the MAEs for the bulk lattice are entirely smaller than those for the training datasets and also comparable to our previous results.^[44,45]^ Although the MAEs for the $\Sigma13$ GB are larger than that of the bulk lattice except for the energies at 400 K, they are still in a similar level to those of the training datasets. Figure S4 shows errors in MD simulations for the Y-segregated systems. It is seen from Figure S5(a) that the bulk lattice does not exhibit critical errors at all Y concentrations and temperatures, as the data points are not far apart from the diagonal line. Figures S5(c) and (d) indicate that the MAEs for the Y-segregated lattice are comparable to those for the pristine lattice. Similarly, critical errors are also absent for the $\Sigma13$ GB, as shown by Fig. 5(b). These results suggest that the NNP has the ability to accurately predict the energetics for the pristine and Y-segregated systems for both the bulk and GB.

It should be noted that the MAEs for the $\Sigma13$ GB are around 2-3 times larger than those for the bulk lattice and vary with the Y concentrations, reaching the maximum values of 15.6 meV/atom and 245.1 meV/Å for the energy and force, respectively, at 37 cat. % of Y and 400 K. Considering that the $\Sigma13$ GB was used as a reference structure for generating the training datasets, the increased MAEs may reflect the limitation of the current NNP to approximate the potential energy surface around GBs. Thus a careful examination would be required when an NNP is used to predict GB properties for which the very high predictive ability is required, e.g., lattice vibrational properties and free energies. Nevertheless, our purpose in this work is to determine the lowest-energy Y configuration at the $\Sigma13$ GB, and it has been successfully achieved by the present NNP, as indicated below and in the main text.

Figure S6 shows the correlation between the GB energies for NNP and DFT calculations for the Y-segregated $\Sigma13$ GB. In this calculation, NNP-based MCMD simulations were performed to predict the lowest-energy atomic structure for a given Y concentration and cell size, as described in the main text. The obtained structures were all used to perform DFT single-point calculations and to obtain their DFT GB energies. The NNP is found to accurately predict DFT values from low to high-energy states. The MAE over all data points is evaluated to be 0.028 J/m^2^, suggesting that the NNP can distinguish an energy difference between Y-segregated GB structures within this error on average. More importantly, the lowest-energy structure for the NNP is the same as that for DFT calculations and is consistent with STEM images, as mentioned in the main text. This highlights that the NNP has enabled us to explore Y-segregated GB structures in low-energy states without direct DFT-based MCMD simulations, which greatly reduces computational cost and accelerates our understanding of GB atomic structures involving segregants.

**Note S4: Selection of MC and MD temperatures**

In our MC simulations, a single trial move was defined as the exchange of the positions of one Al atom and one Y atom. As MC simulations proceed, the energy decreases due to one successful exchange becomes small, typically within about 0.1-0.2 eV. Considering that the potential energy was allowed to fluctuate within around this range, we set the temperature to 1000 K. At this temperature, Equation 1 indicates that energy increases of 0.1 eV and 0.2 eV occur with probabilities of about 0.31 and 0.10, respectively. At higher temperatures, Al and Y atoms are frequently exchanged even when the potential energy substantially increases, which results in slow convergence to low-energy atomic structures. At lower temperatures, only small fluctuations are allowed, which increases the chance of trapping in local minima. MD simulations served as a simulated annealing approach, allowing the structural unit to transform to lower-energy ones. However, energy barriers for transformation between different structural units can be large, depending on their atomic structures and relative grain positions. In MD simulations at 2000 K for the 1 × 1 × 3 supercell with 2 removed Al_2_O_3_ units and 6 Y atoms, the potential energy fluctuated within standard deviations of 6.37 eV, facilitating transformations of structural units. At higher temperatures, atomic structures were sometimes highly disordered, whereas at lower temperatures, transformations of structural units hardly occur due to small energy fluctuations.

**Note S5: Convergence of the MCMD methods and the reliability and adequacy of the sampling**

With our MC protocol, energy changes due to exchanges of Al and Y atoms became sufficiently small at the final stage of most MCMD simulations, as shown in Fig. S7. This indicates that the atomic structure has reached a local extremum with a GB energy lower than that of the initial impurity configuration. Since whether such an extremum corresponds to the lowest GB energy is generally unknown, the GB energies of multiple atomic structures must be compared by performing multiple independent MCMD simulations, as in Figure. 2(a) and 2(b). In this work, 10 independent MCMD simulations were performed. In addition, two GBs introduced by three-dimensional periodic boundary conditions exhibited different atomic structures at the end of most MCMD simulations, as they were independently treated during MD simulations. For each condition, 20 atomic structures were thus compared by reconstructing two supercells each containing one type of the two GBs, as illustrated in Fig. S8. For the 1 × 1 × 3 supercell with 2 removed Al_2_O_3_ units and 6 Y atoms, 3 out of 20 structures corresponded to the lowest-energy atomic structure shown in Fig. 2(c). This supports that this atomic structure corresponds to the globally optimal solution.

**References**

33 Kresse, G. & Furthmuller, J. Efficiency of ab-initio total energy calculations for metals and semiconductors using a plane-wave basis set. *Comp Mater Sci* **6**, 15-50 (1996).

34 Kresse, G. & Furthmuller, J. Efficient iterative schemes for ab initio total-energy calculations using a plane-wave basis set. *Phys Rev B* **54**, 11169-11186 (1996).

35 Blochl, P. E. Projector Augmented-Wave Method. *Phys Rev B* **50**, 17953-17979 (1994).

36 Kresse, G. & Joubert, D. From ultrasoft pseudopotentials to the projector augmented-wave method. *Phys Rev B* **59**, 1758-1775 (1999).

37 Perdew, J. P., Burke, K. & Ernzerhof, M. Generalized gradient approximation made simple. *Phys Rev Lett* **77**, 3865-3868 (1996).

38 Parrinello, M. & Rahman, A. Crystal-Structure and Pair Potentials - a Molecular-Dynamics Study. *Phys Rev Lett* **45**, 1196-1199 (1980).

39 Parrinello, M. & Rahman, A. Polymorphic Transitions in Single-Crystals - a New Molecular-Dynamics Method. *J Appl Phys* **52**, 7182-7190 (1981).

40 Behler, J. & Parrinello, M. Generalized neural-network representation of high-dimensional potential-energy surfaces. *Phys Rev Lett* **98**, 146401 (2007).

41 Behler, J. Atom-centered symmetry functions for constructing high-dimensional neural network potentials. *J Chem Phys* **134**, 074106 (2011).

42 Behler, J. Constructing high‐dimensional neural network potentials: A tutorial review. *International Journal of Quantum Chemistry* **115**, 1032-1050 (2015).

43 Artrith, N., Urban, A. & Ceder, G. Efficient and accurate machine-learning interpolation of atomic energies in compositions with many species. *Phys Rev B* **96** (2017).

44 Yokoi, T., Hamajima, A., Ogura, Y. & Matsunaga, K. Grain boundary segregation of Y and Hf dopants in α-Al_2_O_3_: A Monte Carlo simulation with artificial-neural-network potential and density-functional-theory calculation. *Journal of the Ceramic Society of Japan* **131**, 751-761 (2023).

45 Yokoi, T. *et al.* Atomic and electronic structure of grain boundaries in a-Al_2_O_3_: A combination of machine learning, first-principles calculation and electron microscopy. *Scripta Materialia* **229** (2023).

46 Shah, S., Palmieri, F. & Datum, M. Optimal Filtering Algorithms for Fast Learning in Feedforward Neural Networks. *Neural Networks* **5**, 779-787 (1992).

47 Blank, T. B. & Brown, S. D. Adaptive, global, extended Kalman filters for training feedforward neural networks. *Journal of Chemometrics* **8**, 391-407 (1994).
